# Opinion: Research hotspots and global trends in respiratory syncytial virus over past five years

**DOI:** 10.3389/fmicb.2026.1772495

**Published:** 2026-02-27

**Authors:** Sujing Weng, Qimin Tang, Hua Zhao

**Affiliations:** 1 Hubei University of Chinese Medicine Affiliated Gong'an Hospital of Traditional Chinese Medicine, Jingzhou, China; 2 Jingzhou Hospital of Traditional Chinese Medicine, Jingzhou, China

**Keywords:** bibliometrics, children, research hotspots, respiratory syncytial virus, trends

## 1 Introduction

Respiratory syncytial virus (RSV) is the main cause of acute lower respiratory tract infections in children under 2 years old. In recent years, the exponential growth in biomedical literature has prompted considerable attention to be directed toward bibliometrics as a method capable of quantitatively and qualitatively analyzing research trends and hotspots within a given discipline. We read with great interest the publication by Tao X (2025). [1] titled “Research hotspots and global trends in respiratory syncytial virus over past five years,” which was published in the issue of Frontiers in Microbiology. This study focused on revealing the development trends and research hotspots in the field of RSV over the past 5 years. A comprehensive review of the extant literature identified several areas of research that are currently receiving significant attention: clinical case analysis and disease burden of RSV, epidemiology and transmission patterns, pathogenic mechanisms, antiviral immune responses, antiviral treatments, and vaccine development. International collaboration has been instrumental in propelling advancements in existing vaccine technologies. Through the collective utilization of resources, the future research directions for global health strategies have been further delineated.

## 2 Commentary

We express our profound support and appreciation for the researchers' contributions to this field and extend our sincere gratitude for their efforts. Nevertheless, several issues were identified that required clarification and correction.

Firstly, the abstract states the following: A comprehensive search encompassing 7,238 articles and comments was conducted. However, the text also states, “A total of 7,290 articles were ultimately analyzed from 2020 to 2024.” A total of 7,238 articles and reviews from the past 5 years were retrieved from the SCI-Extended version of WoSCC. It is recommended that authors clearly specify the total number of included literatures and make changes.

Secondly, the search formula is identified in the main text. The search formula in Supplementary File 1 is inconsistent with the following terms: “Respiratory syncytial virus,” “Syncytial virus,” “syncytial-virus,” “syncytial virus,” “RSV,” “RSV virus,” or “orthopneumovirus.” It is recommended that the author provide clarification regarding the final search formula and implement the necessary corrections.

**Figure 1:**
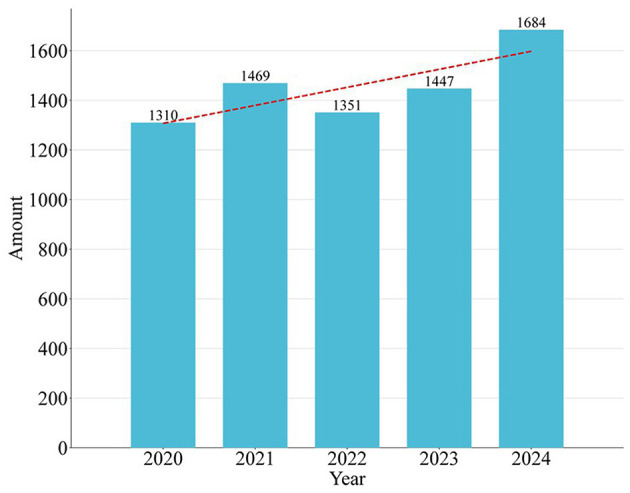
Amount of publications by year during the past 5 years.

Thirdly, the section “Annual trend in the quantity of paper publication” in the text states: The number of yearly papers increased from 1,319 in 2020 to 1,684 in 2024. However, it is important to note that the cumulative number of documents in 2020 was 1,310, as shown in Figure 1. This suggests that there may be a discrepancy between the two figures.

**Figure 2:**
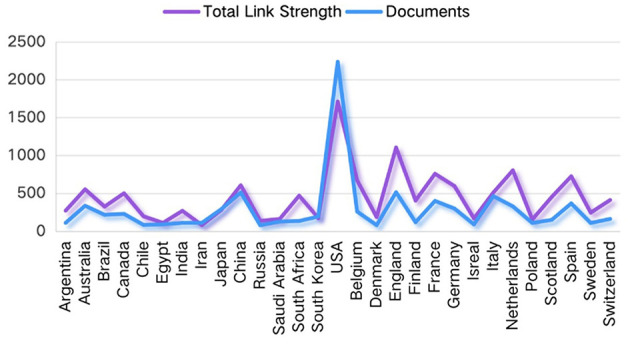
The total link strength and documents of cooperation among the productive countries/regions.

A fourth point of contention is found in the section entitled “Contributions of countries/regions to global publications,” wherein the data concerning the number of publications by several countries is contradictory (Supplementary File 2). For example: As previously documented, the United States published the most articles (2,278/33.1%), followed by China (1,524/22.14%) and England (527/7.65%). However, subsequent documentation revealed that the USA had a higher document (2,236), England (total link strength 1,105, documents 514). A particularly salient example of this phenomenon is evident in Figure 2, wherein the number of articles published by China is clearly no more than 1,000, a figure that is substantially less than the previously cited 1,524.

Fifth, the paragraph entitled “Analysis of authors” states: it is interesting that although Bont, Louis J., who ranks second, has a higher number of articles, he is relatively low in terms of NC (883), H-index (17), and average citation per item (20.71). However, as illustrated in Supplementary File 3, the second row indicates “Bont, Louis J,” and the fifth row indicates. “Bont, L. J,” their countries and institutions are identical. These correspondences suggest a potential identity between the authors. We posit that a combined analysis of the two would be a judicious approach, as it has the potential to more effectively accentuate their academic status within this research domain.

In conclusion, we would like to express our sincere gratitude for the valuable contributions of Xiaoli Tao et al. Their work has not only facilitated the provision of strategic insights to researchers but has also illuminated the path for future research in the domain of RSV research. The identification of the most critical indicators in the field of RSV research would facilitate a more profound comprehension of RSV among researchers and inform decision-making processes. However, to maintain the rigor and scientificity of the article, it would be advisable for the author to employ more rigorous expression.

## Supplementary material

Supplementary File 1 Flowchart of the screening process.

Supplementary File 2 Top 10 countries with the highest productivity.

Supplementary File 3 Top 10 authors with the most publications.
