# Supplementary figures and images for: Opinion: Research hotspots and global trends in respiratory syncytial virus over past five years
Source: Front Microbiol. 2026 Feb 27;17:1772495. doi: 10.3389/fmicb.2026.1772495 (PMC12982400; doi:10.3389/fmicb.2026.1772495)

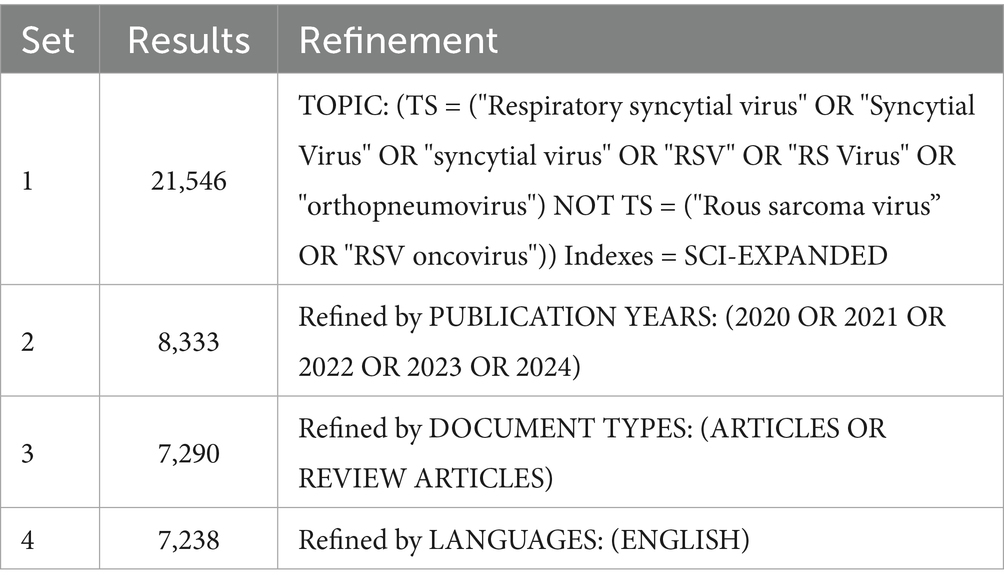

Supplement: Supplementary File 1 — Flowchart of the screening process. [file Image_1.JPEG]

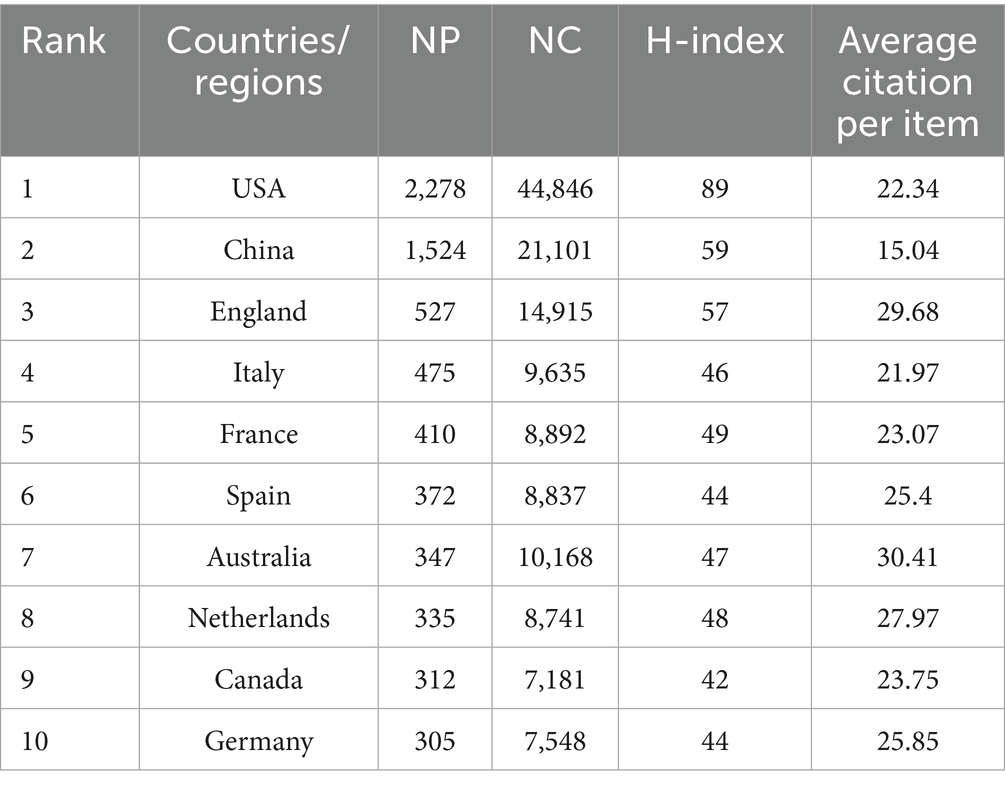

Supplement: Supplementary File 2 — Top 10 countries with the highest productivity. [file Image_2.JPEG]

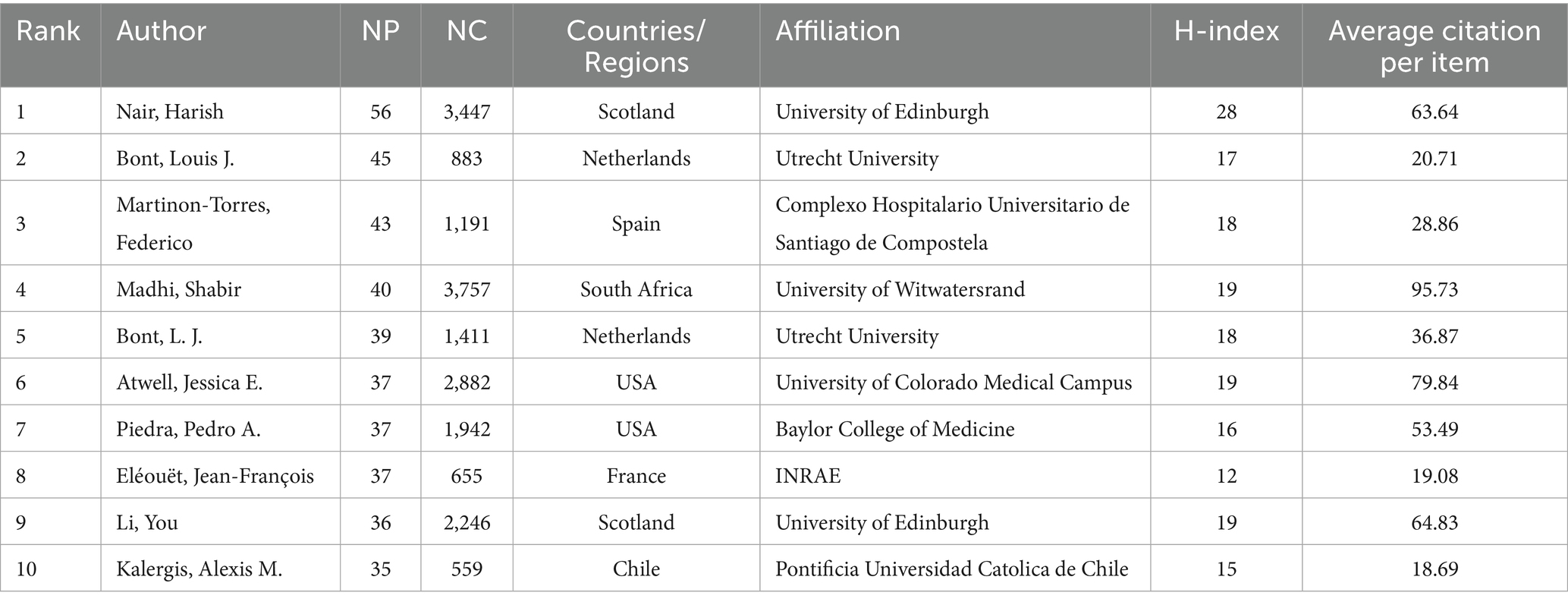

Supplement: Supplementary File 3 — Top 10 authors with the most publications. [file Image_3.JPEG]
